# Data-science based analysis of perceptual spaces of odors in olfactory loss

**DOI:** 10.1038/s41598-021-89969-9

**Published:** 2021-05-19

**Authors:** Jörn Lötsch, Alfred Ultsch, Antje Hähner, Vivien Willgeroth, Moustafa Bensafi, Andrea Zaliani, Thomas Hummel

**Affiliations:** 1 Institute of Clinical Pharmacology, Goethe - University, Theodor Stern Kai 7, 60590 Frankfurt am Main, Germany; 2 Fraunhofer Institute for Translational Medicine and Pharmacology ITMP, Theodor-Stern-Kai 7, 60596 Frankfurt am Main, Germany; 3 DataBionics Research Group, University of Marburg, Hans – Meerwein - Straße, 35032 Marburg, Germany; 4 Smell & Taste Clinic, Department of Otorhinolaryngology, TU Dresden, Fetscherstrasse 74, 01307 Dresden, Germany; 5 Department of Psychology, Lyon Neuroscience Research Center, INSERM U1028 – CNRS UMR5292, Lyon, France; 6 Fraunhofer Institute for Translational Medicine and Pharmacology ITMP/ScreeningPort, Schnackenburgallee 114, 22525 Hamburg, Germany

**Keywords:** Olfactory system, Nutrition

## Abstract

Diminished sense of smell impairs the quality of life but olfactorily disabled people are hardly considered in measures of disability inclusion. We aimed to stratify perceptual characteristics and odors according to the extent to which they are perceived differently with reduced sense of smell, as a possible basis for creating olfactory experiences that are enjoyed in a similar way by subjects with normal or impaired olfactory function. In 146 subjects with normal or reduced olfactory function, perceptual characteristics (edibility, intensity, irritation, temperature, familiarity, hedonics, painfulness) were tested for four sets of 10 different odors each. Data were analyzed with (i) a projection based on principal component analysis and (ii) the training of a machine-learning algorithm in a 1000-fold cross-validated setting to distinguish between olfactory diagnosis based on odor property ratings. Both analytical approaches identified perceived intensity and familiarity with the odor as discriminating characteristics between olfactory diagnoses, while evoked pain sensation and perceived temperature were not discriminating, followed by edibility. Two disjoint sets of odors were identified, i.e., d = 4 “discriminating odors” with respect to olfactory diagnosis, including cis-3-hexenol, methyl salicylate, 1-butanol and cineole, and d = 7 “non-discriminating odors”, including benzyl acetate, heptanal, 4-ethyl-octanoic acid, methional, isobutyric acid, 4-decanolide and p-cresol. Different weightings of the perceptual properties of odors with normal or reduced sense of smell indicate possibilities to create sensory experiences such as food, meals or scents that by emphasizing trigeminal perceptions can be enjoyed by both normosmic and hyposmic individuals.

## Introduction

Olfaction is an important component of quality of life^1–3^. Olfactory deficits reduce the pleasure of eating, influence the relationship with food and increase the risk of accidents in the household^3,4^. Furthermore, reduced or missing sense of smell is associated with depression^5,6^ and has effects on sexual and social life^7^. The perceived loss of olfactory function is a reason for an estimated 80,000 people per year in the German-speaking countries to see a doctor^8^, with an estimated 5% incidence of total loss of olfactory function worldwide^9^. Olfactory function is routinely tested in clinical practice with test batteries that focus on the sensory perception of odors, which is clinically useful and can be applied quickly, especially when using one of their many shortened versions^10–12^. However, this testing reduces the perception of odors to the sensory dimension, which has prompted consideration of other ways to more fully characterize olfactory loss, including measurements of olfactory recognition, identification, sizing, and hedonics^13,14^.

Odor perception is based on the binding of ligands (odorous molecules) to olfactory receptors that are thought to recognize specific molecular features. An important rule for this interaction is that a given odorant can activate one or several odorant receptors. This combinatorial coding is then processed by higher brain structures, resulting in odor percepts. A major challenge is how to integrate the physicochemical properties of odor and its perceptual qualities (e.g., intensity, familiarity, pleasantness, and enjoyability). The physicochemical space of odors is defined by properties such as the type of atoms, the length of the carbon chain, the type of bonds or functional groups, etc.^15^. The perceptual space of odors is defined by the pleasantness^16^, intensity^17^, familiarity^18^, or edibility of the odor source^19^. In addition to olfactory features, irritating or cooling sensations can also be elicited by odors, referring to a "trigeminal perceptual space"^20^.

Hence, the present study aimed to investigate the influence of reduced olfactory sensory function diagnosed by a standard clinical test on other dimensions of olfactory perception. Therefore, subjects with normal or reduced olfactory sensory acuity were asked to evaluate a wide range of different odors with respect to perceptual characteristics, selected based on previous publications on the dimensionality of odors^21–23^. The study addressed the changes in the perceptual space of smell^24^ when olfactory function deteriorates. Its focus was on the question of which dimensions of perception are most or least influenced by the loss of olfactory function and to which odors this applies most often. For the latter, the hypothesis was investigated whether odors with the most or least perceptual changes fall into groups of chemical properties. Regarding the methodological approach, we chose machine learning to provide a relatively unbiased view of the olfactory spaces of patients with loss of olfactory function.

## Methods

### Study setting and design

This was a prospective cohort study performed in a specialized smell and taste clinic. The study was performed in accordance to the Declaration of Helsinki on Biomedical Studies Involving Human Subjects. The study was approved by the ethics committee at the University Clinic of Dresden (approval number EK 390102014). All participants provided informed written consent. All patients were referred or self-referred to the Clinic for Smell and Taste of the Department of Otolaryngology, TU Dresden. Since familiarity and hedonicity are sensitive to cultural factors, caution should therefore be exercised in generalizing the study results for these specific parameters to other cultural contexts.

### Participants

A total of 172 volunteers participated; 106 of them were healthy normosmic subjects recruited via flyers and 66 of them were patients with loss of olfactory function who presented themselves at the Smell & Taste Dysfunction outpatient clinic. Inclusion criteria were age 18 years and older, non-smoking, absence of pregnancy, absence of a neurodegenerative disease such as Parkinson's or Alzheimer's. In addition, at least residual olfactory function was required, i.e., only subjects with normosmia or hyposmia as olfactory diagnoses were included while anosmia, the third of the three commonly accepted olfactory diagnoses, was an exclusion criterion. Causes of olfactory dysfunction included upper respiratory tract infections (n = 28), head trauma (n = 4), sinunasal disease (e.g., chronic rhinosinusitis, nasal allergies: n = 9), idiopathic causes (n = 23), and other causes (n = 2; myasthenia gravis, herpes encephalitis).

All participants underwent a standardized diagnostic procedure that included a detailed, medical history and a detailed physical otorhinolaryngological examination^25,26^. In addition, olfactory function of all participants was assessed using an established clinical test^27,28^ (“Sniffin’ Sticks”, Burghart Instruments, Wedel, Germany), which evaluated three sensory dimensions of odors comprising odor threshold (to phenylethylalcohol), odor discrimination (16 pairs of odors) and odor identification (16 odors). The olfactory functional diagnosis was obtained from the sum of scores for *T*hreshold, *D*iscrimination and *I*dentification (TDI) subtests, with a range between 1 and 48 points and allows to categorize subjects as normosmic (> 30.5), hyposmic (16.5–30.5), and functionally anosmic (< 16.5), based on normative scores obtained in more than 9000 healthy subjects^29^. At the end of the measurements, participants were also asked whether they felt hungry or not. Among patients, 76% reported not feeling hungry, compared to 65% among controls (t.-test: p = 0.11). This indicated that the feeling of hunger was not comparable between the two groups.

### Variables and measurements

#### Perceptual ratings of odors

A total of 40 odorants (Table 1; obtained from Sigma-Aldrich, Taufkirchen, Germany), which cover a wide range of the stimulus space of odor^22^, were dispensed with an air dilution olfactometer^30^. Odors were chosen to represent the multidimensionality of odors, which includes chemical, olfactory, and trigeminal perceptual features. Potentially, there are billions of odorants^31^. Therefore, odorants were selected from standard atlases^32,33^ that reference hundreds of odorants to roughly cover these dimensions.

**Table 1:** Composition of the four odor test sets, including concentrations used and CAS numbers of the chemicals.

Set #	Component	Smell	Concentration [ % vol:vol mineral oil]	CID	CAS-No
1	Isoamylacetat	Banana, pear	0.032	31,276	123-92-2
1	Cineol	Eucalyptus	0.5	2758	470-82-6
1	Geraniol	Fruity, rose	Pure	637,566	106-24-1
1	Methylsalicylat	Bubble gum, wintergreen	7.26	4133	119-36-8
1	trans-Anethol	Liquorice, anise	4.17	637,563	4180-23-8
1	Ethylacetat	Sweet, "pear drops"	10	8857	141-78-6
1	Propionic acid	Stinging, vinegar, acidic	0.041	1032	79-09-4
1	Eugenol	Clove	pure	3314	97-53-0
1	2-Nonanone	Fruity, cheesy	1	13,187	821-55-6
1	Indole	Sweet, unpleasant	0.161	798	120-72-9
2	Benzaldehyde	Marzipan, cherry, almond	0.015	240	100-52-7
2	Butyric acid	Rancid butter, parmesan cheese, vomit	0.001	264	107-92-6
2	p-Cresole	Livestock waste	0.018	2879	106-44-5
2	Guajacole	Band aid, sweet, creamy	2.09	460	90-05-1
2	(+)-Linalool	Lemon, lime	2.17	6549	126-90-9
2	(+)-Fenchone	Minty, camphor-like	pure	1,201,521	4695-62-9
2	HMHA	Sweaty	0.01	16,666,688	58,888-76-9
2	Amyl caproate	Banana, fruity	0.56	31,266	540-07-8
2	2, 3-Butandione	Butter, perspiration	3E-05	650	431-03-8
2	Citronellal	Lemon	0.014	7794	106-23-0
3	cis-3-Hexenol	Grass	0.002	5,281,167	928-96-1
3	1-Butanol	Cheese, sweat	pure	263	71-36-3
3	4-Ethyl octanoic acid	Goaty	pure	61,840	16493-80-4
3	β-ionone	Lilac	7.27	638,014	79-77-6
3	2-Methyl propanal	Wet cereal or straw	1E-06	6561	78-84-2
3	Terpinene-4-ol	Musty	pure	11,230/5,325,830	562-74-3
3	Isobutyric acid	Rancid butter	1	6590	79-31-2
3	4-Decanolid	Peachy	10	12,813	706-14-9
3	Citronellol	Lemony	17.85	8842	106-22-9
3	3-Methyl-3-sulfanylhexan-1-ol	Sweaty	0.01	10,130,039	307964-23-4
4	D-(+)-Limonene	Lemony	pure	440,917	5989-27-5
4	alpha-Pinene	Woody, pine, resinous	pure	440,968	80-56-8
4	Methional	Potato	0.001	18,635	3268-49-3
4	Benzyl acetate	Yasmin, fruity, ylang	1.55	8785	140-11-4
4	1-Octen-3-ol	Mushrooms	0.56	18,827	3391-86-4
4	trans-2-Hexenyl acetate	Fruity, apple, waxy	10	17,243	2497-18-9
4	L-Carvone (−)	Caraway	pure	439,570	6485-40-1
4	Beta-Caryophyllen	Peppery, spicy, resinous	pure	5,281,515	87-44-5
4	Heptanal	Fruity, sharp	1	8130	111-71-7
4	2-Butanone	Cheese	0.01	6569	78-93-3

Experiments in a panel of 10 experienced subjects, trained before the actual experiments, ascertained that the odors presented during the main experiment were of similar intensity. For this purpose, odorous substances were diluted in propylene glycol if necessary (vol/vol concentrations in the liquid phase). Subsequently, the odors were presented using the specially designed computer-controlled olfactometer^34^, at a total flow rate of 2 l/min (Table 1). Four sets of 10 stimuli each were used, ensuring that each participant was randomly tested with a single set (Supplementary Fig. S1). Each odorant was presented birhinally through a flexible polyurethane tube that reached about 1 cm into the nasal cavity to release odors beyond the nasal valve. An additional nasal cannula (AirLife™, tube with 2.8 mm inner diameter) was used to monitor breathing (AWM2100V, Honeywell, MN, USA), so that olfactory stimuli of 5 s duration were emitted at the beginning of an inspiration phase. The order of the odorants was random. Each odor was presented in three repetitions at an interval of 40–60 s. The sequence of odor presentations was also randomized for each of the three blocks of presentations.

After each stimulus presentation, the participants were asked to rate seven different perceptual properties of the odors, at a randomized order comprising edibility, intensity, irritation, temperature, familiarity, hedonics, and painfulness, selected based on previous publications on the dimensionality of odors^21–23^. These dimensions were coded with grades from 1 to 5. The ends of the scales were labeled with the following (left hand end—right hand end): *Edibility* (“how much would like to eat something that smells like this”: “not at all”—“very much”), *intensity* (“how intense is the odor”: “barely perceptible”—“very intense”), *irritation* (“how irritating do you find the odor”: “not at all irritating”—“very irritating”), *temperature* (“how cool/warm do you find the odor”: “very cool”—“very warm”), *familiarity* (“how familiar are you with the odor”: “not familiar at all”—“very familiar”), *hedonics* (“how much do you like the odor”: “very unpleasant”—“very pleasant”) and *pain* (“how painful do you find the odor”: “not painful at all”—“very painful”). Each odor was rated twice on a computer monitor. Thus, the odors were presented 3 times, but not all ratings were made after each stimulus, so that in the end there were, for example, 2 ratings for each of the perceptual properties.

### Data analysis

#### Quantitative variables

The data set initially originally included n = 173 subjects and d = 4 × 10 × 7 = 280 variables. The variables resulted from the design of the study where 4 different sets of 10 different odors each were rated with respect to 7 properties including edibility, intensity, irritation, temperature, familiarity, hedonics and painfulness. For each subject, the data set contained an additional variable that carried the olfactory diagnosis of normosmia or hyposmia. Missing odor ratings were imputed for subjects who had performed at least two thirds of the required evaluations, using k-nearest neighbors (kNN) with k = 3 within the respective olfactory set, calculated with the R-library "DMwR". (https://cran.r-project.org/package=DMwR^35^). In addition, it was examined whether the subjects assigned to the four separate odor sets showed equal distributions of age, sex and odor diagnoses, using Kruskal–Wallis^36^ and χ^2^^37^ tests, respectively. However, due to the small sample size per odor set and olfactory diagnosis, they were not considered further.

#### Assessment of the significance of perceptual odor ratings for olfactory diagnosis

To obtain an internal validation of the findings, two separate approaches were used to analyze whether the property evaluations of odors provide differences among normosmic and hyposmic subjects. *Firstly*, the input space was submitted to *unsupervised* analyses implemented as a projection method (principal component analysis, PCA^38,39^). This was aimed at detecting structures that supported a separation of the olfactory diagnoses. *Secondly*, the data space was submitted to *supervised* analysis where a machine-learning based classifier was trained with the task to find a mapping of the odor ratings to the olfactory diagnoses. If the trained classifier performed better than guessing on data not used for training, it can be assumed that the ratings of olfactory characteristics contain relevant information for the olfactory diagnosis. The analyzes were performed using the R software package (version 4.0.3 for Linux; https://CRAN.R-project.org/^40^ on an Intel Core i9® (Intel Corporation, Santa Clara, CA, USA) computer running Ubuntu Linux 20.04.1 LTS 64-bit (Canonical, London, UK)).

##### Unsupervised analysis of odor property ratings for structures reflection olfactory diagnostic groups

**Figure 1:**
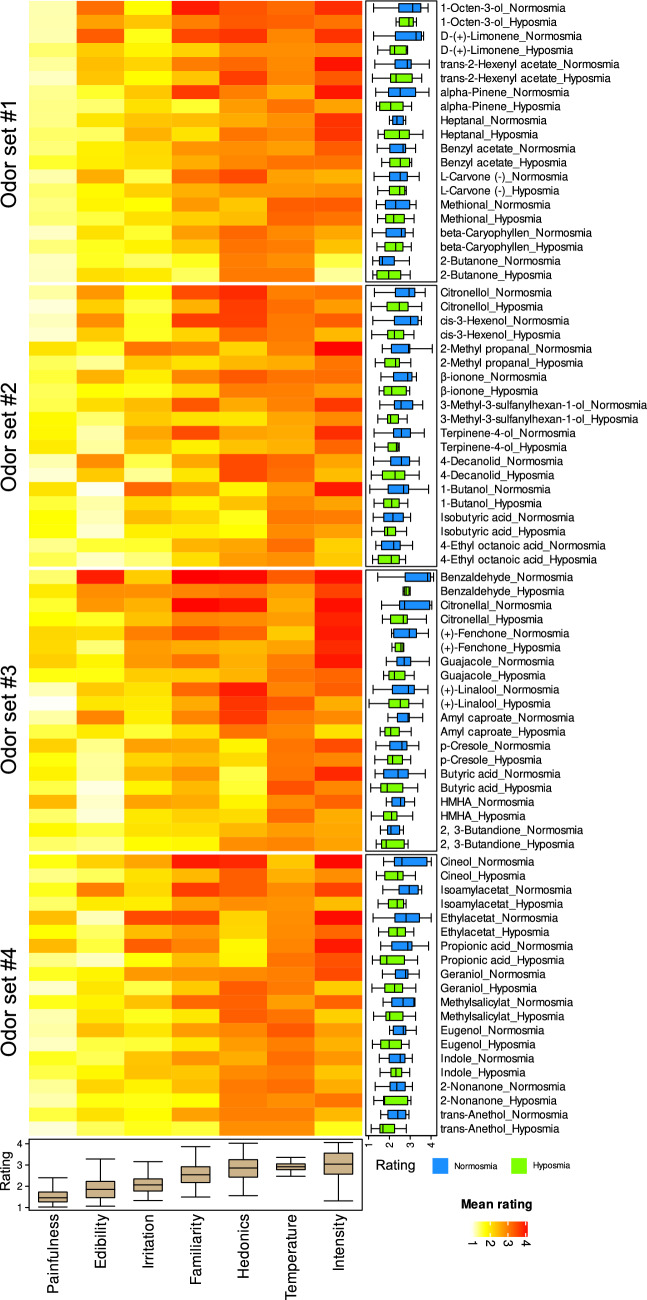
Heat plot of the means of the ratings of odors (rows) for seven different properties (columns). The 40 odors used in the study were split into four sets of 10 odors each (indicated at the left). The matrix is sorted column wise, per odor set, to locate the lowest ratings at the bottom left corner and the highest ratings at the upper right corner. Marginal statistics are shown as boxplots, displaying the minimum, quartiles, median (solid line within the box) and maximum. The whiskers add 1.5 times the inter-quartile range (IQR) to the 75th percentile or subtract 1.5 times the IQR from the 25th percentile. Outliers and extreme values are omitted from the boxplots; therefore, please note that the scale of the axis includes scores [0,…,4], while the data range is [0,…,5] as indicated in the methods description section. The figure has been created using the R software package (version 4.0.3 for Linux; https://CRAN.R-project.org/^40^), and the R library “ComplexHeatmap” (https://bioconductor.org/packages/release/bioc/html/ComplexHeatmap.html^68^).

To analyze whether the olfactory property ratings had structural features that supported the separation between the olfactory diagnoses, ratings were averaged separately for each of the 7 properties and olfactory diagnoses. This resulted in an 80 × 7 matrix (Fig. 1), i.e., 40 odors, which were evaluated by either normosmic or hyposmic subjects with respect to the seven perceptual properties. These perceptual data were projected onto a two-dimensional space using PCA on non-scaled data and the default settings of the R-base method "prcomp". The results were analyzed for the significance of olfactory properties for group separation between normosmic and hyposmic subjects, based on the loadings of the evaluated properties on the relevant principal components (PCs). As PCA identified two principal components which explained 85.5% of the total variance, projection was done on a two-dimensional space defined by the two most relevant PCs. A measure for group separation was obtained by the odor-specific Euclidean distances between paired normosmic and hyposmic cases. Finally, to select the relevant odors, an item categorization technique was implemented as computed ABC analysis^41^, which partitions a set of positive values into three disjointed subsets called “A”, “B” and “C”^42^. The subset “A” contains the few most relevant items^43^. These calculations were done using our R package “ABCanalysis” (https://cran.r-project.org/package=ABCanalysis)41.

##### Supervised analysis of odor property ratings for information allowing to separate olfactory diagnostic groups

Four different sets of 10 odors each, which were evaluated with respect to seven characteristics by four different sets of participants, provided a 146 × 280 sparse block diagonal matrix (odor properties) with ratings for olfactory characteristics, specifically 4 sets of 10 odors each and 7 queries per odor = 280 columns, one for each odor and property (Supplementary Fig. S1). To this added the class information regarding the two olfactory diagnoses. To assess whether the property ratings of odors provided information relevant for the separation of normosmic and hyposmic subjects, a supervised machine-learning algorithm in form of random forests^44,45^ was trained to map the odor properties for the 146 subjects) onto the two olfactory diagnoses. Random forests were built using the R library “randomForest” (https://cran.r-project.org/package=randomForest)^46^, with hyperparameters set at 1,000 trees with $0.5\cdot \sqrt{n_{features}}$ and a maximum of seven nodes per tree, which was established in a grid search. As it is known that more trees do not confer a risk of increasing errors^47^, a larger number was considered safe and merely consumed available computation time. Classifier training was performed using 1,000 runs on 2/3 of the data (training subset) obtained by Monte-Carlo^48^ resampling from the original data, using the R-library "sampling" (https://cran.r-project.org/package=sampling)^49^. The resampling was adjusted such that the diagnoses of normosmia and hyposmia and the subjects tested with each of the four odor sets were represented at equal proportions.

During each run, the importance of each odor rating was measured as the mean decrease in the Gini impurity if the respective feature was excluded from random forest learning. To assess whether the random forests were able to detect differences between normosmic and hyposmic subjects, as a prerequisite for using feature importance as a quantitative measure of the significance of each rating, the trained classifiers were applied to the one third of the data not used for training during each run. The classification performance was evaluated by calculating the area under the ROC curve (AUC-ROC) using the R-library “pROC” (https://cran.r-project.org/package=pROC)^50^. The classification performance was again assessed using permuted data for training with the expectation that then the classification will be not better than guessing, else overfitting was likely. In addition, during each run the values of *–log(p)* resulting from Wilcoxon-Mann–Whitney-U tests^51,52^ for group differences between the olfactory diagnoses were kept, for which the actual training data subsets were used. Both measures were averaged for the 1,000 runs and provided finally two 40 × 7 sized matrices (40 odors, seven rated perceptions). These performance measures were rank transformed. A combination under independence assumption is calculated by a multiplication of the two measures. That is, we merged the importance in random forests, measured as the decrease in Gini impurity when the particular feature was omitted when training the forest, with the degree of statistical difference, measured as Wilcoxon W. The direct way to merge such differently scaled measures was to rank-transform them and combine the ranks. Here, multiplication implies a logical "AND", i.e., a feature is considered important if it is important to the performance of the random forest algorithm AND produces a comparatively larger statistical effect size. Finally, the relevant odors were selected using computed ABC analysis^41^ as described above. By applying the rank transformation of above-mentioned matrices the opposite direction, a similar ABC analysis provided the least important odors and properties for the separation of odor diagnoses.

##### Combination of the unsupervised and supervised results

For the calculation of the combined sets of odor and perceptual characteristics which can either distinguish or not between the two olfactory diagnoses the results from the unsupervised and supervised analyses were intersected.

#### Assessment of specific chemical properties of odors relevant for the olfactory diagnosis

The two lists of odors, which either distinguish between the two olfactory diagnoses or have no property relevant for this distinction, were analyzed for chemical differences. The underlying hypothesis was that a common receptor family could be responsible (e.g., a G-protein coupled receptor (GPCR) subfamily). The focus was narrowed down to one of the simplest groups of pharmacophore-based descriptors called Chemically Advanced Template Search (CATS) 2D^53^, considering the possibility to retain structural information when coding a molecular graph according to the reciprocal bond distances of atom pairs. The atom pairs under consideration were all possible combinations (10) of five different atom types (L = lipophilic, A = acceptor, D = donor, N = negatively charged, P = positively charged). These pharmacophoric atom types reflect the interaction types that are possible for a ligand with a protein counterpart. The necessary molecular descriptors of chemical properties^54^ were obtained using the Dragon software (version 6, Talete s.r.l., Milan, Italy; http://www.talete.mi.it, accessed on May 22, 2020). This provided n = 90 features of the CATS class. The maximum distance considered for counting the internal binding distance was eight, due to the size of the molecules of current interest. The total number of possible symmetrical combinations is (n*(n − 1)/2) i.e., 5*4/2 = 10 and the distance = 8 (9 distances), summed up to 10 × 9 columns, which formed the chemical feature matrix used. A statistical model was derived that could help to classify the two odorant quantities and predict future ones. Nevertheless, the number of independent variables was extended to other known pharmacophore-based descriptors such as MOE2D^55^ or RDKit^56^ (see also https://www.rdkit.org/UGM/2012/Landrum_RDKit_UGM.Fingerprints.Final.pptx.pdf, accessed on June 8, 2020); however, without finding any real benefit in this extension, which were therefore not included in the final analyses. Tree-based classification models were trained, including random forests and hierarchical classification and regression trees implemented as bagged CART^57^. All models were established by leave-one-out cross-validation due to the small size of the odor sets, with positive control scheme and challenged with random permutation of the dependent variables as negative control. The classification performance was assessed as described above using the AUC-ROC.

## Results

### Participants and descriptive data

At least two-thirds of the odor property assessments were available from n = 146 subjects (median: 93.75% of the complete data per subject) who provided the analyzed cohort. An overview of the mean scores per odor and perceptual property is shown in Fig. 1. The examined persons were between 18 and 82 years old (mean value ± standard deviation: 41.1 ± 18.6 years). For 38, 33, 42 and 33 subjects, odor sets 1–4 were used, each comprising 8, 9, 17 and 8 hyposmic patients.

The age of the subjects was similar for the odor sets (Kruskal–Wallis χ^2^ = 4.065, df = 3, p = 0.2545). The distribution of normosmic and hyposmic subjects (χ^2^ = 4.2794, df = 3, p = 0.2328) and of both sexes (χ^2^ = 32.9841, df = 3, p = 0.3941) was also similar for the odor sets.

### Main results

#### Significance of perceptual odor ratings for the olfactory diagnosis

##### Unsupervised analysis of odor property ratings for structures reflection olfactory diagnostic groups

**Figure 2:**
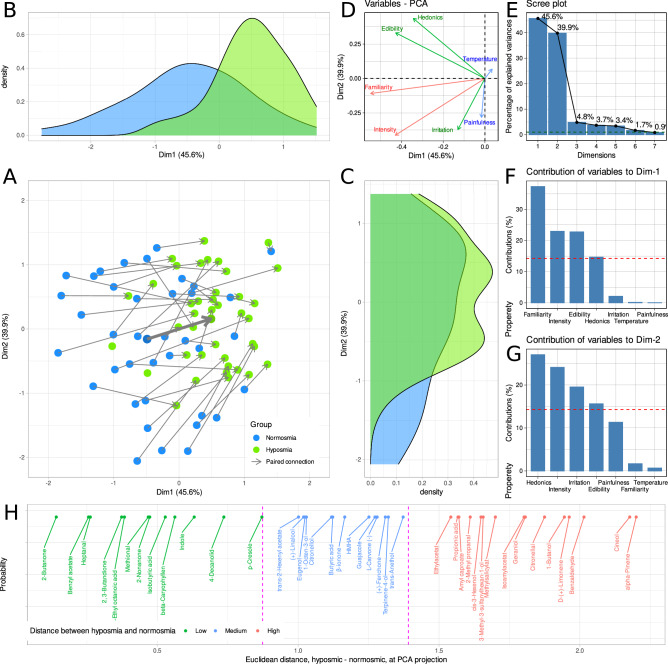
Results of a principal component analysis (PCA). Projection of the 80 × 7 data matrix obtained by averaging the ratings of perceptual odor properties, separately for each of the seven properties, the 40 odors and the 2 olfactory diagnoses. (**A**) Plot of the data projected on the space given by the first two principal components (Dim.1 versus Dim.2). The PCA plot shows the separation of olfactory diagnoses mainly to the right in Dim.1 and to the top in Dim2, see the thick arrow indicating the averages of the PCA coordinates between olfactory diagnoses. The same odors rated by either normosmic or hyposmic subjects are connected with arrows (paired data). (**B,C**) The marginal distribution plots show the segregation of the pain phenotype groups along the principal components. (**D**) Plots the Eigenvectors of a variable in PCA Dim1 versus Dim2. (**C**) Scree-plot of the amount of variance of the data captured by each principal component. (**E**) Bar graph of the explained variance by each principal component. (**F**) Bar graph of the contribution of each perceptual property to Dim.1. The dashed horizontal reference dashed corresponds to the expected value if the contribution where uniform. (**G**) Bar graph of the contribution of each perceptual property to Dim.2. (**H**) Sorted Euclidean distances between the same odors evaluated by either normosmic or hyposmic subjects, i.e., the lengths of the arrows in panel A. The vertical dotted lines show the decision boundaries obtained by ABC analysis of the distances. The figure has been created using the R software package (version 4.0.3 for Linux; https://CRAN.R-project.org/ (R Development Core Team, 2008)) and the libraries “ggplot2” (https://cran.r-project.org/package=ggplot2 (Wickham, 2009)) and “FactoMineR" (https://cran.r-project.org/package=FactoMineR^69^).

The first two principal components (PC) obtained in the PCA (Fig. 2) of the differences between hyposmic and normosmic subjects in the ratings of 40 different odors with respect to seven different perceptual characteristics (Fig. 1) explain more than 85 percent of the total variances (Fig. 2E). The main differences between olfactory diagnoses were observed in the direction of PC1 (Dim1 in Fig. 2), with familiarity, intensity and edibility contributing most to PC1 (Fig. 2F). The ratings of pain sensation, temperature and irritation perceived in the presentation of odors were the most important along PC2 (Dim2 in Fig. 2). ABC analysis of the Euclidean distances between the projections of the mean ratings of each odor by normosmic or hyposmic subjects on the PC1 x PC2 plane (Fig. 2A–C) the three sets of 12, 13 and 15 odors with small, medium and large distances between odor diagnoses (Fig. 2F). These borders were consistent with large gaps in the sorted distances (Fig. 2F). The extreme groups were interpreted as odors that were non-distinctive (n = 12, marked green in Fig. 2H) or distinctive (n = 15 marked red in Fig. 2H) between the two odor diagnoses.

##### Supervised analysis of odor property ratings for information allowing to separate olfactory diagnostic groups

**Figure 3:**
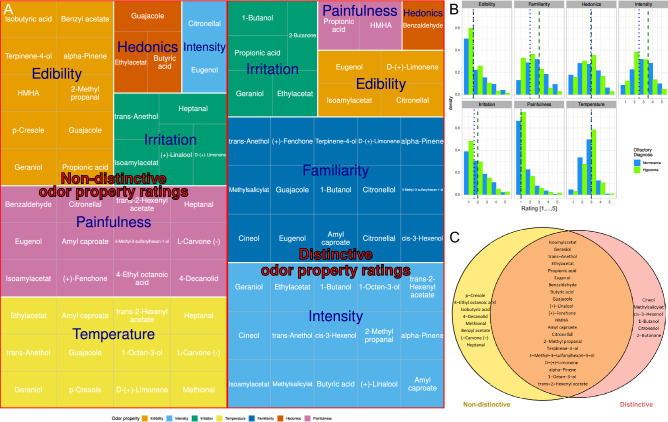
Perceptual ratings of odors that possess properties that are informative for the distinction between normosmic and hyposmic subjects, or that lack such properties. (**A**) Tree map of odor property assessments. The figure is a structured representation of the results of the item categorization analysis in a hierarchical order. The first level represents the grouping of the odor property ratings in terms of the information they provide for distinguishing normosmic from hyposmic subjects. The subsequent levels show perceptual properties and odors separated from each other. The second level represents the perceptual properties. The size of the rectangles corresponds to the number of odors that possess the respective property in connection with the separation of normosmic and hyposmic test persons. The third level represents the individual odors that possess the respective property that has been found to be informative or non-informative for the separation of olfactory diagnoses. (**B**) Histograms of the pooled ratings of seven properties for all odors. The dotted and dashed perpendicular lines mark the medians of the respective ratings, separately for normosmic (dashed) and hyposmic (dotted) subjects. (**C**) Venn diagram showing the sets observed between two groups of odors identified to represent characteristics that best distinguish between olfactory diagnoses or, on the other hand, are least distinctive between olfactory diagnoses (Table 2). The figure has been created using the R software package (version 4.0.3 for Linux; https://CRAN.R-project.org/^40^) and the R libraries RAM” (https://cran.r-project.org/package=RAM^70^), “ggplot2” (https://cran.r-project.org/package=ggplot2^71^) and “treemapify” (https://cran.r-project.org/package=treemapify^72^).

The trained random forest classifier used on the individual ratings of 40 odors with respect to seven different properties (Supplementary Fig. S1) was able to correctly assign subjects to the olfactory diagnosis of either normosmia or hyposmia with a median AUC-ROC of 73.6% (95% confidence interval 52.8–90.3%), observed during 1000 cross-validation runs. When trained with randomly permuted data, the median AUC-ROC was 54.2%, which can be considered as close to pure guessing. Subsequently, the feature importance in the random forest classifier was rank-transformed and multiplied with the rank transformed statistical group differences (normosmic versus hyposmic subjects). An ABC analysis of these ranks provided in subset “A” n = 42 odor property ratings (Supplementary Fig. S2) containing n = 27 different odors and all seven perceptual properties (Table 2). However, by far most often the ratings of the odor’s familiarity and perceived intensity were distinctive between the olfactory diagnoses (Fig. 3A). This also captured the properties that across all odors were rated most differently by normosmic or hyposmic subjects (Fig. 3B). For the inverted ranks, the respective results were n = 44 odor property ratings containing 29 odors and seven properties. There, painfulness and intensity were the most frequently occurring properties, followed by edibility (Table 2, right part). Due to the inverse ranking, this denoted the least distinctive properties between the odor diagnoses. The two sets of odors partly overlapped, which led to two disjoint sets of d = 6 odors that had only property ratings exclusively discriminating and d = 8 that had only property ratings exclusively non-distinguishing between the olfactory diagnoses could be separated (Fig. 3C).

**Table 2:** Odors identified to represent characteristics that best distinguish between olfactory diagnoses or, on the other hand, are least distinctive between olfactory diagnoses.

Most relevant for the olfactory diagnosis				Least relevant for the olfactory diagnosis			
1-Butanol	3	Intensity	15	Guajacole	3	Temperature	12
Isoamylacetat	2	Familiarity	15	Heptanal	3	Painfulness	12
Cineol	2	Irritation	5	Isoamylacetat	2	Edibility	10
Geraniol	2	Edibility	4	Geraniol	2	Irritation	5
Methylsalicylat	2	Painfulness	2	Trans-anethol	2	Hedonics	3
Trans-anethol	2	Hedonics	1	Ethylacetat	2	Intensity	2
Ethylacetat	2			Eugenol	2		
Propionic acid	2			p-Cresole	2		
Eugenol	2			Amyl caproate	2		
Amyl caproate	2			Citronellal	2		
Citronellal	2			D-(+)-limonene	2		
Cis-3-hexenol	2			Trans-2-hexenyl acetate	2		
D-(+)-limonene	2			L-carvone (-)	2		
Alpha-pinene	2			Propionic acid	1		
Benzaldehyde	1			Benzaldehyde	1		
Butyric acid	1			Butyric acid	1		
Guajacole	1			(+)-Linalool	1		
(+)-Linalool	1			(+)-Fenchone	1		
(+)-Fenchone	1			HMHA	1		
HMHA	1			4-ethyl octanoic acid	1		
2-Methyl propanal	1			2-methyl propanal	1		
Terpinene-4-ol	1			Terpinene-4-ol	1		
Citronellol	1			Isobutyric acid	1		
3-Methyl-3-sulfanylhexan-1-ol	1			4-Decanolid	1		
1-Octen-3-ol	1			3-Methyl-3-sulfanylhexan-1-ol	1		
Trans-2-hexenyl acetate	1			Alpha-pinene	1		
2-Butanone	1			Methional	1		
				Benzyl acetate	1		
				1-Octen-3-ol	1		

The table shows the items assigned to ABC set “A” by computed ABC analyses of the rank products of odor property importance for the olfactory diagnosis. The left part of the table shows the most relevant odors and properties in descending order of occurrence in ABC set “A”. The right part of the table shows the opposite analysis, i.e., aiming at the odors and properties that were least distinctive between the olfactory diagnoses of normosmia versus hyposmia. The original data consisted of an odor and a rated perceptive property. The numbers in the table show how many times an odor or a perceptive property was assigned to ABC set “A”, i.e., to the set of most relevant items.

##### Combination of the unsupervised and supervised analyses results

Finally, both lines of data analysis were used to derive odors that have perceptual properties that are most or least differently assessed by hyposmic subjects compared to subjects with normal olfactory function. PCA and machine learning based analysis consistently identified the perceived intensity and familiarity with the odor as the distinguishing characteristics between olfactory diagnoses. Evoked pain sensation and perceived temperature were identified as non-distinguishing characteristics, i.e., similarly evaluated independently of their olfactory functional performance, followed by the assessment of edibility. These results are also supported by the median ratings of the perceived olfactory properties given by hyposmic subjects compared to subjects with normal olfactory function (Fig. 3B).

The odors that were identified in the supervised analysis as being perceived by hyposmic subjects either differently or similarly to normosmic subjects were also identified in the unsupervised analysis at 66.7% and 77.7% respectively. The intersection of the results of the two approaches resulted in the final set of n = 4 "distinctive odors" consisting of cis-3-hexenol, methyl salicylate, 1-butanol and cineol, while the final set of n = 7 "non-distinctive odors" included benzyl acetate, heptanal, 4-ethyl-octanoic acid, methional, isobutyric acid, 4-decanolide and p-cresol (Supplementary Fig. 3).

### Other results: chemoinformatics explorations

#### Specific chemical properties of odors relevant for the olfactory diagnosis

**Figure 4:**
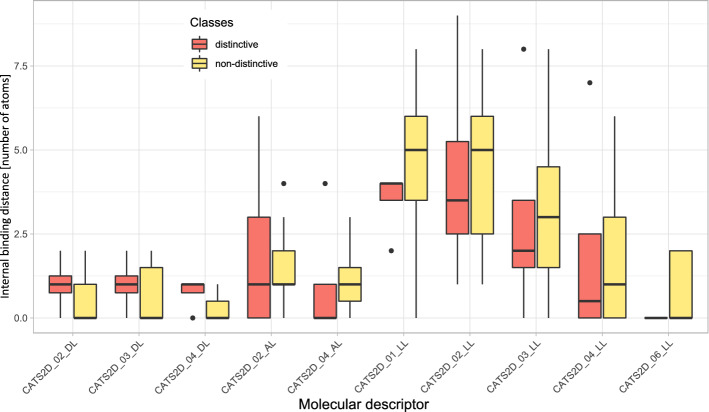
Results of the exploration of differences between distinctive and non-distinctive odors with respect to chemically Advanced Template Search (CATS) 2D molecular descriptors. The boxplots of the top ten CATS2D variables used in random forests and bagged classification and regression tree models. The boxes have been constructed using the minimum, quartiles, median (solid line within the box), and maximum. The whiskers add 1.5 times the inter-quartile range (IQR) to the 75th percentile or subtract 1.5 times the IQR from the 25th percentile. The figure has been created using the R software package (version 3.6.1 for Windows; https://CRAN.R-project.org/^40^) and the R library “ggplot2” (https://cran.r-project.org/package=ggplot2^71^). (*L* lipophilic, *A* acceptor, *D* donor, *N* negatively charged, *P* positively charged).

For the disjoint sets of d = 4 exclusively discriminating and d = 7 exclusively non-discriminating odors, 90 molecular CATS2D descriptors were initially used (such ligand-based chemical similarity approaches have been effectively applied, for example to predict the activity of drugs^58^). Those with a variance < 0.2 or internal correlations > 0.75 were removed. The names of the CATS2D descriptors are coded as follows: “CATS2D_”, “distance2D_”, “type atom pair”. Thus, “CATS2D_06_AL” means: the count of all molecular graph distances = 6 between atom pairs acceptor-lipophilic (AL) (Fig. 4).

The machine-learning algorithms trained with the remaining 20 descriptors, i.e., random forests (RF) and bagged CART achieved high performances with leave-one-out cross-validation of median AUC-ROC = 100%, which substantially exceeded the values obtained when the training data were permuted (61 and 48% respectively). The tree-based models were further analyzed to extract the most contributing descriptors, i.e., the most important variables used by the trained algorithm to perform the classification. This resulted in the identification of CATS2D_04_DL, CATS2D_03_DL and their similar CATS2D_XX_LL descriptors for distance = 2–3.

## Discussion

The preserved or reduced olfactory function is reflected differently in the perception of different odorous substances. Based on the demonstration of the suitability of olfactory property assessments that allow a trained machine-learning algorithm to distinguish between normosmic and hyposmic subjects, this study identified particularly relevant odors and related subjective characteristics. A key finding of the analysis was, that odor properties reflecting trigeminal sensations, such as painfulness and temperature, play the least important role in distinguishing normosmic from hyposmic subjects. On the other hand, familiarity and perceived intensity of odorants seem more to reflect olfactory sensations and are therefore rated higher by persons with normal olfactory function than by persons with a reduced sense of smell.

### Discussion of main results

In fact, the perceptual characteristics that differ between normosmic and hyposmic individuals provide information on how odors are perceived by both groups. When distinguishing between normosmic and hyposmic individuals, the perceptual characteristics provide relevant information in the order of familiarity, odor intensity, irritation, edibility, hedonics and painfulness. In contrast, of the two olfactory diagnosis groups of subjects, the most similar ones were evaluated in the order lack of distinct information, painfulness, temperature, edibility, irritation, hedonics and odor intensity. From this it can be deduced that in normosmic subjects, familiarity with odors plays an important role in perception. This perception disappears with a decreasing sense of smell, leaving the prevailing trigeminal sensations that are predominant in the perception of odors. The present results thus indicate a shift up to a partial reversal of olfactory perception when the olfactory function deteriorates. This finding could be used in the creation of food or fragrances. While such efforts are common in many clinical pictures that attempt to alleviate disease-related restrictions in the daily lives of patients, similar efforts for the benefit of hyposmic individuals are still rare. A deeper insight into the shifts in the perceptual characteristics of odors is the rational basis of such efforts. In particular, the present results highlight the significance of the "trigeminality" of odors, which could be more fully exploited in the development of foods or fragrances for people with impaired sense of smell (which includes the large group of elderly people who typically exhibit subtle loss of olfactory function^59^). Furthermore, it appears that certain odors are less affected by the loss of olfaction than others.

Seven of originally 40 odorous substances were identified by two independent approaches as the best differentiation between normosmic and hyposmic subjects. These substances did not have any perceptual properties and did not differ between the two diagnostic groups of subjects. This set of apparently predominantly olfactory stimulations included cineol, 2,3-butanedione, cis-3-hexenol, 1-butanol, terpinene-4-ol, citronellol and 2-butanone. They smell of eucalyptus, butter, cut grass, sweat/cheese, resin, lemon, and butterscotch. Because the distinction between olfactory diagnoses seemed to become more difficult when trigeminal properties were involved, these odorants qualify for inclusion in olfactory tests. So far, current tests only partially cover this set. For example, butanol is used in the original set of the test battery of Sniffin' Sticks to determine the odor threshold^27,28^ or the Connecticut Chemosensory Clinical Research Center Test for olfactory evaluation (CCCRC^60^).

The grouping of odors into "trigeminal" and "olfactory" stimuli is somewhat problematic. This is mainly because almost all smells produce trigeminal sensations, albeit to varying degrees^61,62^. Furthermore, trigeminal activation increases with odor concentration and stimulus duration^63,64^. A further dimension of the complexity of trigeminal activation arises from the interactions between the trigeminal and olfactory systems^20^ that are modified by olfactory loss^65^. These interactions may help to explain why some of the characteristic odors are often mentioned in connection with trigeminal activation, e.g., cineol^61^—while some of the non-characteristic odors are more on the olfactory side, e.g., methional, a "potato like" odor. However, it has to be kept in mind that most of the previous studies on interactions between the trigeminal and the olfactory systems were based on relatively selective trigeminal stimulus CO_2_. This was different in the present study where odors were used which are typically met in foods, fragrances etc., adding everyday-life aspects to the interaction between the two intranasal systems which the mentioned paper did not provide.

### Discussion of additional chemoinformatics explorations

Boosted classification and regression trees consistently showed as the top three descriptors of the Chemically Advanced Template Search (CATS) 2D type, i.e., "CATS2D_04_DL", "CATS2D_02_LL", "CATS2D_03_DL". These molecular descriptors had higher values for the distinctive odors regarding relevant perceptual characteristics for the assignment of a subject to the olfactory diagnosis of normosmia or hyposmia. Since the chosen internal bond distance counts were 2 to 4, the structural information refers to the spatial position of these atomic pairs (DL and LL with L = lipophilic, D = donor) at the given distances. Usually, local branching or bifurcations within the molecular graphs with small moieties like methyl or ethyl have a profound effect on CATS2D, as they increase all the counts for smaller distances, as here for distances 2 to 4. In addition, this has profound effects on the three-dimensional conformations available to the molecules, since branching always reduces the flexibility of the molecules^66^, which must be accommodated within the cavity of the seven transmembrane helices of the olfactory G protein-coupled receptors (GPCR), which are responsible for the recognition of the odorant and for its signal to the brain. In this case the major effects on model performances have been the counts of lipophilic atoms to hydrogen-donor atoms (OH in our cases). This might suggest for instance that compounds containing secondary or tertiary hydroxyls can be more distinctive than those containing primary alcohols or acid. Even if we cannot exclude the effect of the odorant on other receptor families which might be relevant for olfactory perception (e.g., ion channels), the validity of the description of the ligand pharmacophore remains unchallenged.

### Strengths and limitations

A strength of the analysis is that results were obtained using two different and independent analysis approaches that yielded largely consistent results. The PCA-based analysis examined structures in the data that supported separation of odor diagnoses. In contrast to that, the machine learning approach aimed to use the information in the odor ratings as if the diagnosis were to be made from these assessments. Thus, machine-learning was used without trying to develop a diagnostic tool with maximum classification performance but rather for identifying the most relevant features. A weak point of this approach was the small sample size. In other words, although an apparently quite large cohort with n = 146 subjects was analyzed, the division of the study design into 4 sets and two olfactory diagnoses resulted in a group size of 8 subjects per set and diagnosis. This was the minimum size across the four olfactory sets and determined the sizes of the training data sets, which were chosen so that each olfactory set was represented by the same size. This prevented validation of the machine learning results in a hold-out data set that should have been separated from the data before analysis. An independent validation data set in which the results were reproduced was not part of the present project. To have balanced group sizes for all subsequent analyses, all sets were adjusted to the smallest set or group sizes. This ensured that the results were not dominated by an accidentally larger set, nor was such error hidden in the results. Other approaches would be to use bootstrapping with increase in set sizes or to create further “cases” bay adding white noise to the data, i.e., jittering. However, we did not expect any improvement by these methods on the results obtained. The exploratory design of the present study must be taken into account when interpreting the results. Because the design is novel, a clear sample size calculation could not be made in advance; therefore, a sufficiently powered study will only be possible based on the present results and remains a future task in assessing the role of different olfactory spaces in the context of impaired olfaction.

The resulting sets of distinctive and non-distinctive odors nevertheless represent a converging point from independent lines of data analysis. However, the subsequent investigation of the systematic chemical differences between the two groups of odor molecules was based on very small groups of odor molecules. It is therefore recommended to substantiate chemical differences in odors that are perceived differently or similarly by hyposmic persons compared to persons with normal olfactory function in independent experiments. Finally, a finer stratification of the subjects according to the TDI score of the sensory olfactory performance was not performed due to this limitation of the sample size.

## Conclusions

In this study, the perceptual properties of odorous substances showed structural differences in PCA projection methods and could be used to train a machine-learning algorithm to separate individuals with normal olfactory function from individuals with reduced olfactory function far better than by random association. This provided a basis for assessing which perceptual characteristics of which odors played a role in the successful performance of the machine learned classifier. The main result of this analysis was that as the olfactory function deteriorated, the familiarity of the odors was lost and replaced by the predominant perception of trigeminal sensations such as the sensation of heat or coolness of the odor. This could be associated with two unrelated sets of seven odors each, including an apparently predominantly olfactorily mediated set of odorants which possess properties that are perceived relevantly differently by normosmic persons compared to hyposmic persons, and another set which apparently lacked such properties. Further investigations provided hints that this grouping of odorants has a chemical basis, probably in the number of binding distances of different atomic types in lipophilic pharmacophores or those defined as hydrogen-bonding acceptors or hydrogen-bonding donors^67^. The observed shift in the pattern of olfactory perception from familiarity to trigeminal perception, together with evidence of a chemical basis, can be used in efforts to create fragrances or foods suitable as disability inclusion aimed at people with impaired olfactory function.

## Supplementary Information

Supplementary Information.

## Data Availability

Data available on request from the senior author.
