# Supplementary material for: Data-science based analysis of perceptual spaces of odors in olfactory loss
Source: Sci Rep. 2021 May 19;11:10595. doi: 10.1038/s41598-021-89969-9 (PMC8134481; doi:10.1038/s41598-021-89969-9)
Supplement: Supplementary file 1 — Supplementary Information. [file 41598_2021_89969_MOESM1_ESM.docx]

Data-science based analysis of perceptual spaces of odors in olfactory loss

Jörn Lötsch^1,2^, Alfred Ultsch^3^, Antje Hähner^4^, Vivien Willgeroth^4^, Moustafa Bensafi^5^, Andrea Zaliani^6^, and Thomas Hummel^4^

^1^ Institute of Clinical Pharmacology, Goethe - University, Theodor Stern Kai 7, 60590 Frankfurt am Main, Germany

^2^ Fraunhofer Institute for Translational Medicine and Pharmacology ITMP, Theodor-Stern-Kai 7, 60596 Frankfurt am Main, Germany

^3^ DataBionics Research Group, University of Marburg, Hans – Meerwein - Straße, 35032 Marburg, Germany

^4^ Smell & Taste Clinic, Department of Otorhinolaryngology, TU Dresden, Fetscherstrasse 74, 01307 Dresden, Germany

^5^ Department of Psychology, Lyon Neuroscience Research Center, INSERM U1028 – CNRS UMR5292, Lyon, France

^6^ Fraunhofer Institute for Translational Medicine and Pharmacology ITMP, Schnackenburgallee 114, 22525 Hamburg, Germany

**Correspondence to:** Prof. Dr. Dr. Jörn Lötsch, Goethe - University, Theodor - Stern - Kai 7, 60590 Frankfurt am Main, Germany, e-mail: [j.loetsch@em.uni-frankfurt.de](mailto:j.loetsch@em.uni-frankfurt.de), Phone: +49-69-6301-4589, Fax: +49-69-6301-4354

# Supplementary information

Supplementary Figure 1: Overview of the sparse data matrix used in the supervised analysis. The matrix shows the data setting where separate groups of subjects, comprising always both normosmic and hyposmic subjects, rated separate sets of 10 odors each, without overlap among subjects and odor sets. The lines display the cases, with arbitrary case numbers on the right, and the columns display the features, coded always as odor “_” and rating, i.e., edibility, intensity, irritation, temperature, familiarity, hedonics, and painfulness.


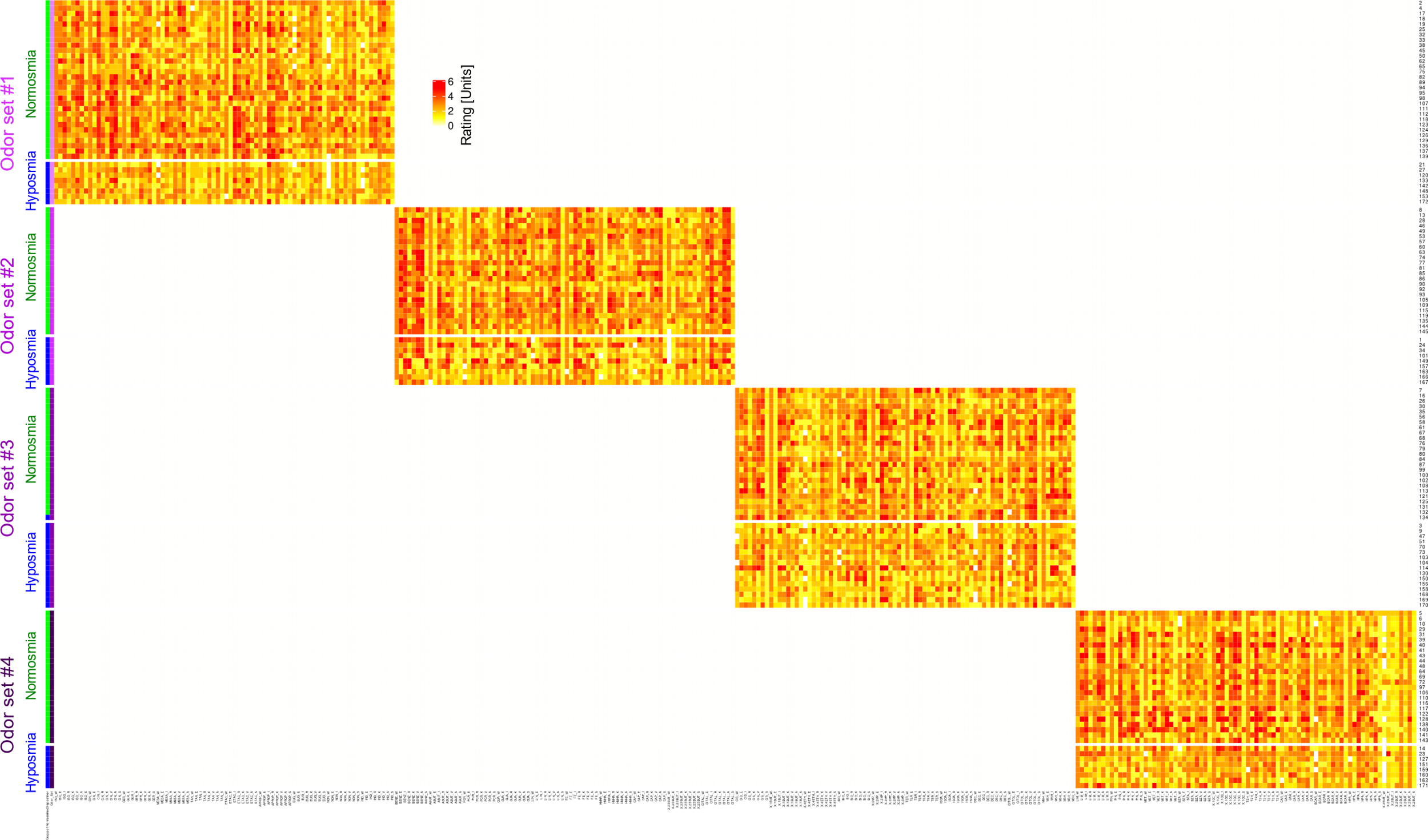


Supplementary Figure 2: Selection process of the odor properties that are most informative for the assignment of a subject to the olfactory diagnosis of normosmia or hyposmia. **A:** Histogram of the distribution of the product of the ranks of the values of the mean decrease in the accuracy of random forest classification when the characteristic was omitted from training and the ranks of the values of *-log10(p)* obtained by conducting paired group comparisons using Wilcoxon-Mann-Whitney-U tests. **B** ABC plot (blue solid line; for details, see ^1^) obtained with the rank products from panel A. The limits of sets A, B and C resulting from the present ABC analysis are drawn as red lines. In addition, ABC plots of the identity distribution, *x_i_* = constant (magenta line), and of the uniform distribution in the data range *U[min(xi),max(xi)]* (green line) are shown for comparison. **C:** Results of a further ABC analysis in the sense of a nested computed ABC analysis, i.e., submitting ABC set “A” of a first analysis shown in Panel B to a second ABC analysis. **D:** Bar chart of the odor property ratings found in ABC set “A”, i.e., among the most important items for the assignment of a subjects to the olfactory diagnosis of normosmia or anosmia. The figure has been created using the R software package (version 4.0.3 for Linux; <http://CRAN.R-project.org/> ^2^) and the R libraries “ABCanalysis” (<http://cran.r-project.org/package=ABCanalysis> ^3^) and “ggplot2” (<https://cran.r-project.org/package=ggplot2> ^4^).


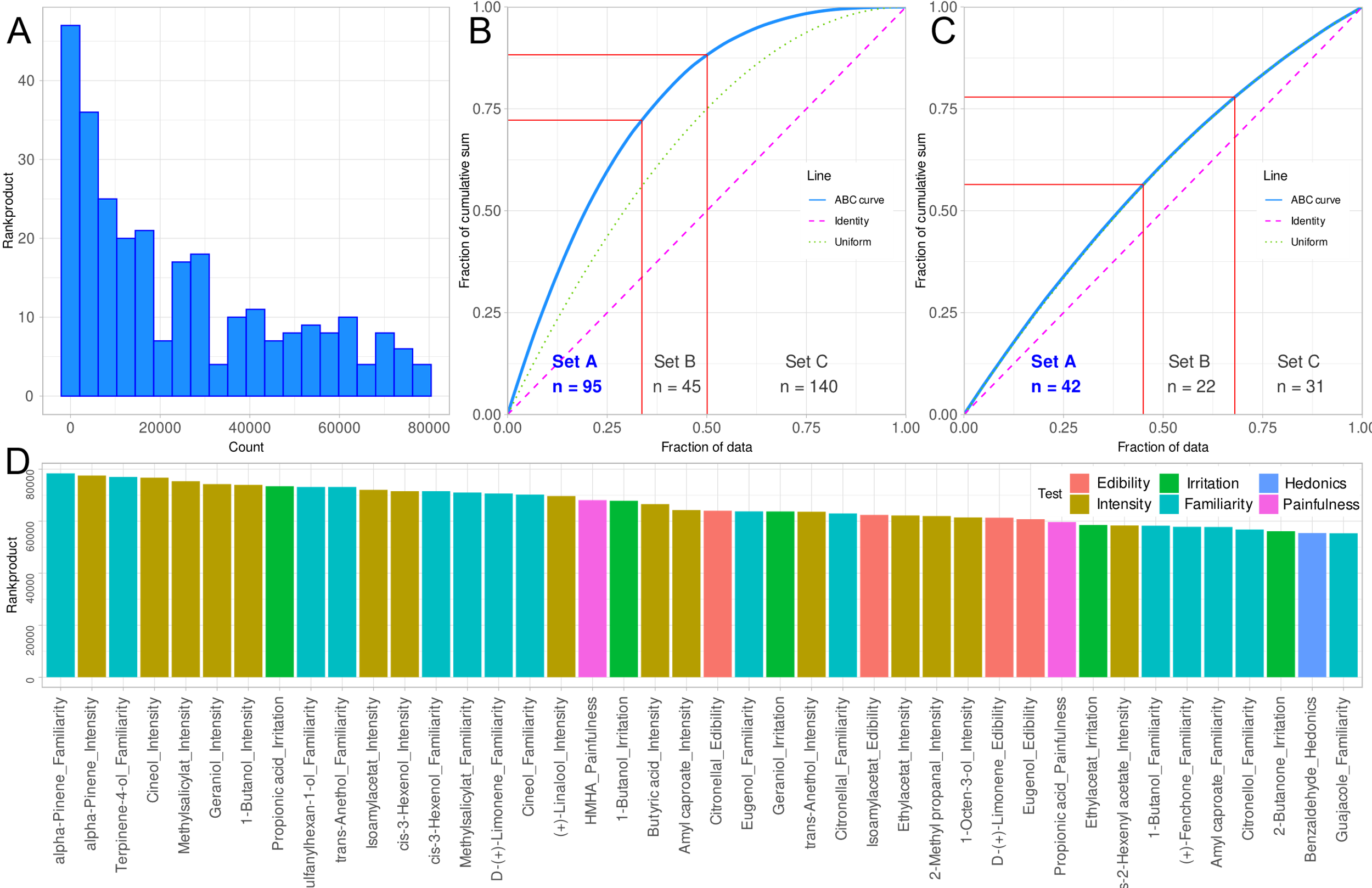


Supplementary Figure 3: Perceptual ratings of odors that possess properties that make them informative for the distinction between normosmic and hyposmic subjects, or that lack such properties. Property ratings of odors that had a discriminating property for the olfactory diagnosis and odors that had no discriminating property for the olfactory diagnosis. Ratings of eight odor characteristics, separately for normosmic persons and patients with hyposmia. The top line shows the ratings of the odors identified as possessing characteristics that best distinguish between olfactory diagnoses. The bottom two rows, on the other hand, show the ratings of odors that were identified as being the least distinctive between olfactory diagnoses. The boxes have been constructed using the minimum, quartiles, median (solid line within the box), and maximum. The whiskers add 1.5 times the inter-quartile range (IQR) to the 75^th^ percentile or subtract 1.5 times the IQR from the 25^th^ percentile. The figure has been created using the R software package (version 4.0.0 for Linux; <http://CRAN.R-project.org/> ^2^) and the R library “ggplot2” (<https://cran.r-project.org/package=ggplot2> ^4^).


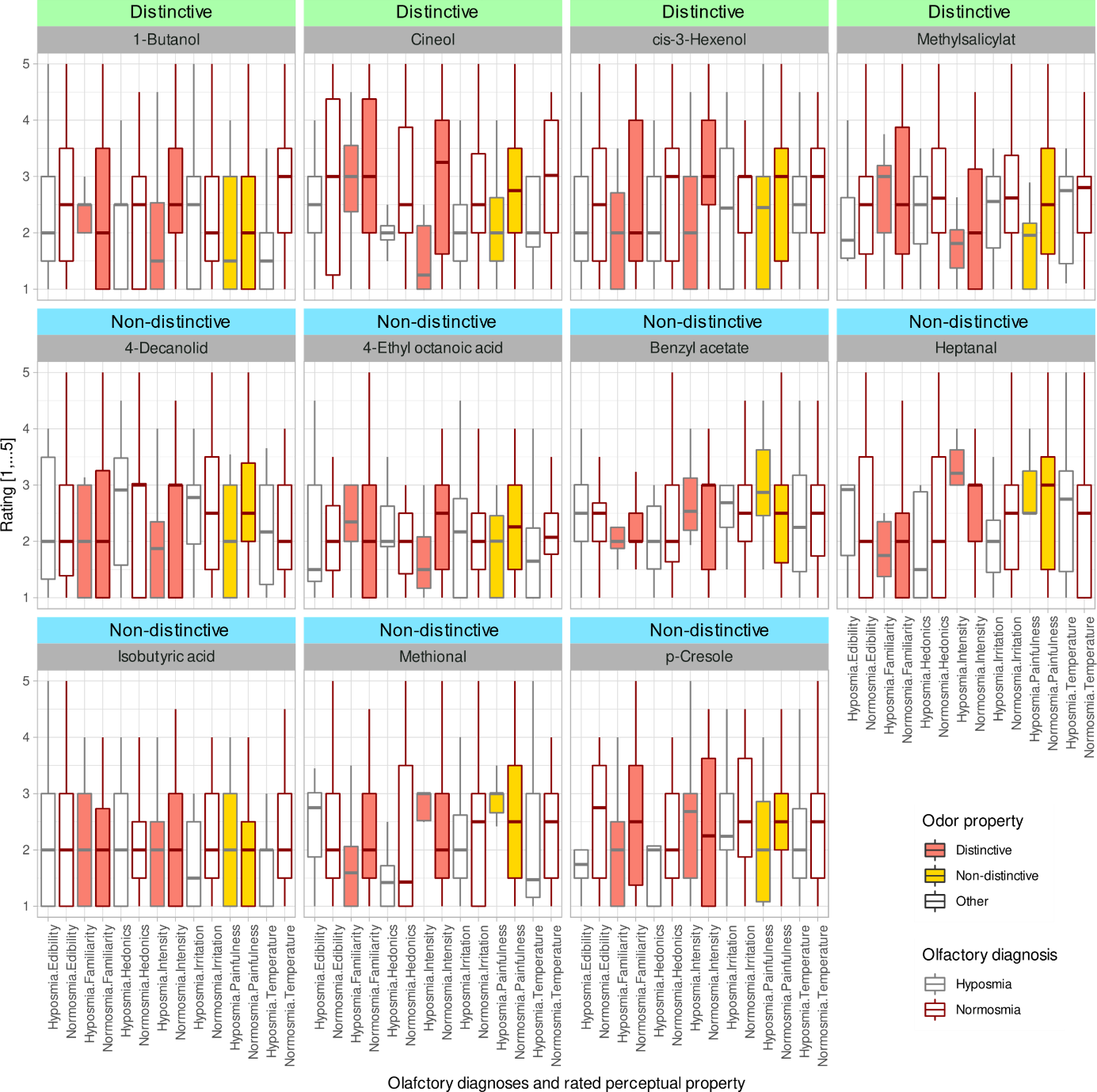


# References

1 Ultsch, A. & Lötsch, J. Functional abstraction as a method to discover knowledge in gene ontologies. *PLoS One* **9**, e90191 (2014).

2 R Development Core Team. R: A Language and Environment for Statistical Computing. (2008).

3 Ultsch, A. & Lötsch, J. Computed ABC Analysis for Rational Selection of Most Informative Variables in Multivariate Data. *PLoS One* **10**, e0129767 (2015).

4 Wickham, H. *ggplot2: Elegant Graphics for Data Analysis*. (Springer-Verlag New York, 2009).
